# Endometrial Cyst Presenting as a Vague Abdominal Lump in a Postmenopausal Woman

**DOI:** 10.7759/cureus.29807

**Published:** 2022-10-01

**Authors:** Poosapati D Devasilpa Raju, Yashwant Lamture, Swati G Deshpande, Rajesh G Gattani

**Affiliations:** 1 Department of General Surgery, Jawaharlal Nehru Medical College, Datta Meghe Institute of Medical Sciences, Wardha, IND

**Keywords:** serous cystadenoma, cea level, tumour markers, endometriosis surgery, ovarian cyst

## Abstract

A postmenopausal woman presented with a predominantly right-sided abdominal lump, insidious in onset and not associated with any aggravating or relieving factors. Physical examination revealed a soft cystic mass extending from the right hypochondrium to the right iliac fossa region crossing the midline. Ultrasonography of the abdomen and pelvis showed a large cystic anechoic area noted in the abdomen extending from the epigastric region to the pelvis. Contrast-enhanced CT of the abdomen and pelvis showed a large non-enhancing cystic lesion in the pelvis suggesting the possibility of a right ovarian cyst or mesenteric cyst. Laparotomy was performed and the excised specimen was sent for histopathological analysis, which confirmed it to be an endometrial cyst.

## Introduction

Though endometriosis prevalence decreases to 2.55% in postmenopausal women [1], endometriosis is a common benign yet painful gynaecological condition affecting about 5-10% of the reproductive age group females [2,3]. The presence of functional endometrial tissue and stroma outside the uterine endometrial cavity defines the condition [2]. Most patients have few or most symptoms in the reproductive years and they rarely present primarily in the postmenopausal age group. Clinical features include irregular bleeding, dyspareunia, dysmenorrhea, increased chronic pelvic pain, and infertility [2]. The endometrial deposits are mostly located in the ovaries, posterior cul-de-sac, uterosacral ligament, broad ligament, bladder, and sigmoid colon [2]. The pathophysiology is complex and multifactorial with contributions from various genetic, epigenetic, hormonal, and immunologic factors [2]. A transvaginal ultrasound scan is usually used as the first choice to examine the ovarian cyst with several advantages including wide availability, lack of exposure to radiation, cost efficiency, and well tolerance [4]. Rarely, contrast-enhanced CT (CECT) abdomen and MRI abdomen are done to rule out malignancy. However, sometimes the differential diagnosis is difficult to make preoperatively. Radical surgery removing all endometrial tissue is the primary therapeutic option [3]. In this case, we are reporting a rare issue of a post-menopausal woman with an endometrial cyst presenting as a vague abdominal lump.

## Case presentation

A 53-year-old P2L2 (para 2 live 2) postmenopausal female presented with swelling in the right side of the abdomen which was insidious in onset and progressive in nature and not associated with any aggravating or relieving factors. There were no bowel or bladder complaints, and no history of weight loss or loss of appetite. She had no significant history of taking OCP or any hormone replacement therapy. General physical examination revealed a soft cystic mass felt was extending from the right hypochondrium to right lumbar region and right flank. All routine lab investigations done were normal. Many differential diagnoses including a mesenteric cyst, abdominal wall lipoma, desmoid tumour, hepatoma, hernia and a large retroperitoneal tumour were kept in mind.

Radiological findings

**Figure 1:**
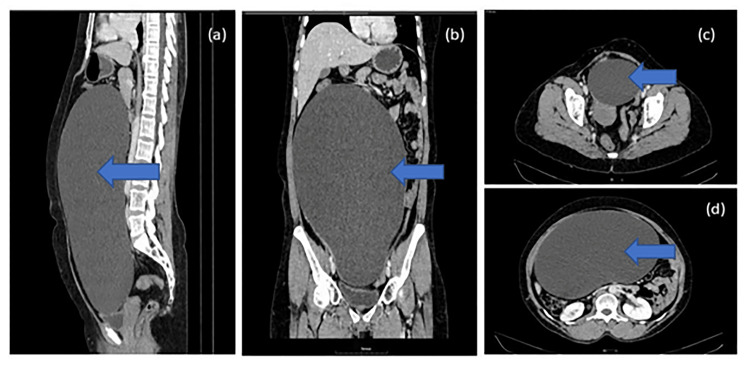
Contrast-enhanced CT (CECT) of abdomen and pelvis showing a) sagittal image, b) coronal image, c) transverse image pelvic region, d) transverse image upper abdomen

Ultrasonography of the abdomen and pelvis showed a large cystic anechoic area noted in the abdominal cavity extending from the epigastric region up to the pelvis, possibly peritoneal. The right ovary was not visualized separately from the lesion. CECT of the abdomen and pelvis (Figure 1) showed a large non-enhancing cystic lesion without any solid component measuring approximately 30cm (CC) x 13cm (AP) x 20 cm (TR) in the pelvis suggestive of a right ovarian cyst or a mesenteric cyst.

The patient's serum carcinoembryonic antigen (CEA) was found to be 1.4 ng/ml (0 to 2.5ng/ml) and cancer antigen 125 (CA125) was 12 units/ml (0 to 35 units/ml). Based on clinical and radiological findings with negative tumour marker levels, a benign cystic lesion of the ovary, ovarian cyst, or mesenteric cyst was considered. The patient was posted for exploratory laparotomy for excision of the cyst.

Intra-operative findings

**Figure 2:**
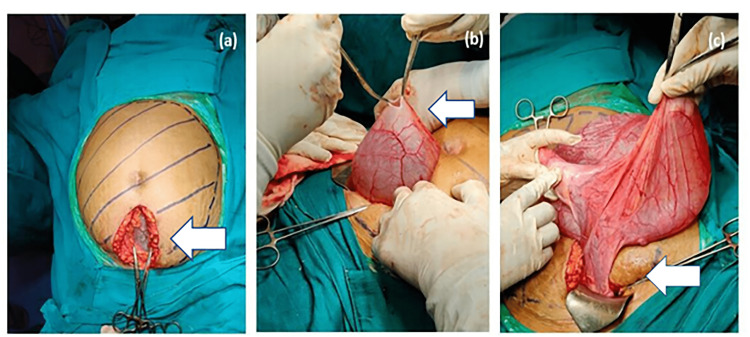
Pictures showing the exploratory laparotomy performed for excision of cystic mass. (a) Blue marking shows the extent of the cyst involving the abdomen and a vertical midline incision was taken extending from the umbilicus to the pubic symphysis to expose the wall of the cyst at its lower end close to the pelvis. (b) Small incision was taken over the cyst wall to aspirate the fluid to decompress the cyst slowly, making it easy to deliver the cyst from the small incision. (c) Decompressed cyst was delivered from the same incision, which was identified as a right ovarian cyst with the right fallopian tube along with mesorchium.

A vertical lower midline incision was taken extending from the umbilicus to the pubic symphysis and the abdomen was opened (Figure 2). A bluish-coloured cyst was identified, which was then examined manually by the operating surgeon, and was found to be involving the whole abdomen extending from the pelvis to the epigastrium, with no adhesions to surrounding structures with probably the right ovary as the origin. Clear straw-coloured fluid was aspirated with a 10 ml syringe and sent for malignant cytology and culture sensitivity. A small slit was taken close to the base of the cyst to evacuate the fluid with precautions to avoid any spillage of contents. Clear serous fluid (5L) was slowly evacuated to decompress the cyst while the anaesthetist monitored it to prevent sudden cardiovascular collapse. The decompressed cyst was then examined and was confirmed to be of right ovarian origin. Complete excision of the cyst with right salpingo-oophorectomy was done.

**Figure 3:**
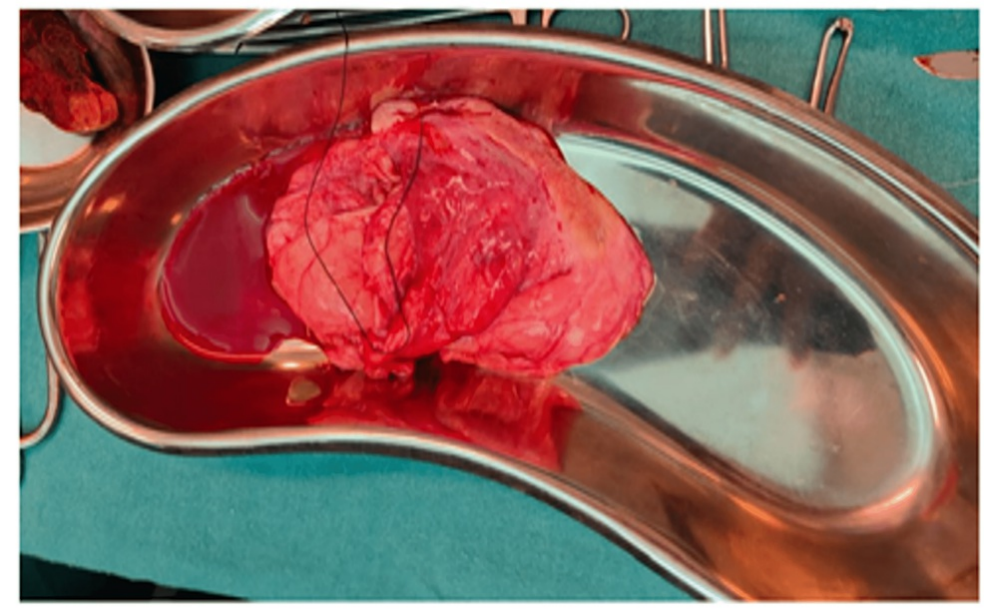
Excised cyst (after the complete evacuation of fluid)

Post-procedure the resected cyst was sent for histopathology (Figure 3) and confirmed to be an endometrial cyst/chocolate cyst of the right ovary.

Post-operative care in the form of regular vital monitoring, IV antibiotics, antipyretics, analgesics, proton pump inhibitors, antiemetics, and a regular sterile dressing was given. The patient was discharged from the hospital once she was clinically and vitally stable with a healthy suture line with advice to follow-up in surgery OPD for further follow-up after one month or SOS (if needed) in case of emergency. 

## Discussion

Postmenopausal endometriosis 

Postmenopausal endometriosis though rare has special renewed interest due to its propensity for malignant transformation [2,4]. Postmenopausal endometriosis is considered to have more complex pathophysiology compared to only excess estrogen exposure in premenopausal endometriosis. It is still unknown whether it is a spectrum of previous premenopausal disease or arises de novo in the postmenopausal period. The predominant estrogen found in postmenopausal patients is estrone, produced by peripheral conversion of androgens. When the estrogen threshold, i.e., when a certain estrogen level, is reached or surpassed in postmenopausal patients, it activates silent foci of endometriosis. In addition to peripheral estrogen production, a high circulating level of exogenous estrogen, especially in the form of phytoestrogens and hormone replacement therapy (HRT) can contribute to the condition [5-7]. Our patient did not have a history of exogenous estrogen or HRT. Previous studies reported that patients with ovarian cysts presented with diffuse abdominal pain and distension, palpable pelvic mass, anorexia, mechanical discomfort, and increased frequency of micturition [8,9]. Sometimes, ovarian cysts may reach huge dimensions without any symptoms as in the above case. In this context, our patient had no classic symptoms except for an abdominal lump that was noticed three months before presenting it to the hospital. However, it was also reported that there is no correlation between the severity of symptoms and clinical presentation.

Also, there are no defined biomarkers for endometriosis. Hence, this indicates that thorough medical history, physical examination, and clinical evaluation can aid clinicians to determine postmenopausal endometriosis only to a limited extent. The differential diagnosis of vague abdominal lump includes a range of conditions such as lipoma, hernia, desmoid tumour, mesenteric cyst or primary or metastatic malignancy [10]. Although a thorough history with proper interpretation of radiological findings might aid in making an accurate preoperative diagnosis, a final diagnosis is made using intraoperative and histopathology findings. In the Asaolu et al. study, an ultrasound scan of the abdomen and pelvis showed a massive uninoculated cystic lesion with clear aqueous content and a dependent layer of hyperreflective echoes. Later, the CT scan erroneously reported a huge unilocular abdominopelvic mass from the ovaries. However, exploratory laparotomy and histopathological analysis confirmed the cyst was affixed to the uterus fundus by a thick peduncle [11].

Hence, surgical removal of cysts and histopathological confirmation is the gold standard diagnostic method for endometrial cysts. In this case, transvaginal ultrasonography was performed initially to identify the cyst and later CT was conducted to rule out cystic lesion vs over distended stomach. Evidence of a large non-enhancing cystic lesion in the pelvis extending up to the liver measuring approximately 30cm x 13cm x 20 cm probably arising from the right ovary suggested the possibility of an endometrial cyst or a mesenteric cyst. We used histopathological analysis to confirm the diagnosis of the endometrial cysts. In a similar study by Chandra et al., premenopausal women admitted with pelvic mass underwent abdominal cystectomy and histopathological examination of excised section confirming the diagnosis of ovarian endometriosis [10]. Although surgery (either diagnostic laparoscopy or excision) is a promising method, non-invasive biomarkers with high sensitivity and specificity are needed to be detected for the diagnosis of endometriosis in the future. The primary treatment of postmenopausal endometriosis is mainly surgical as was first reported in 1950. It was reported that surgical procedures are employed in asymptomatic cysts > 4 cm in diameter and especially in patients > 35 years [3]. Medical management with the use of gonadotropin-releasing hormone (GnRH) analogues, danazol, and progesterone appear to be ineffective in postmenopausal endometriosis [12]. Aromatase Inhibitors targeting peripheral conversion of androgens to estrone may be a new promising method, which could potentially improve symptoms and treat these patients either as a first-line treatment when surgery is contraindicated or as a second-line treatment for recurrences following surgical treatment. However, their use is precluded by side effects like impairment of bone mineral density and an increase in the rate of bone fractures, hence need to be supplemented with bis-phosphonate therapy.

Pharmacotherapy

Pharmacotherapy is considered to play a secondary role during the postoperative period in a few situations, for example, in women with incomplete resection of endometriotic foci or in patients with concomitant pain postoperatively [3]. Commonly used agents include estrogen-progestin preparations, progesterone-releasing intrauterine devices and GnRH agonists. However, in the above-mentioned case, there was no requirement for pharmacotherapy [3].

Risk of malignancy 

Sampson et al., 1925, first described the malignant transformation in endometriosis with an incidence of 1% [13]. The risk of malignant transformation of endometrioma into ovarian cancer is currently estimated at 2% or 3% [14,15]. The endometrial cyst can develop into an endometrioid, clear cell and low-grade serous types. The risk is higher in postmenopausal women with a long-standing history of ovarian endometriosis [16,17]. Kobayashi et al. stated that ovarian endometriomas that are greater than 9 cm in diameter have a strong risk prediction for the development of ovarian cancer in postmenopausal women 45 years of age or older [18]. Endometriosis-related malignancy mostly presents with pressure symptoms, abdominal bloating, and occasionally even gastrointestinal bleeding [2]. However, such findings weren’t seen in the above-described case. Hence, clinicians should include endometriosis in the differential diagnosis of any symptomatic postmenopausal patient with vague abdominal lump with gastrointestinal findings. If endometriosis is confirmed, a proper follow-up of such women is necessary on a long-term basis to prevent future adverse outcomes. Therefore, postmenopausal women with endometriosis must go in for surgical excision to prevent the development of future malignancies [2]. A study by Bertelsen et al. included 115,000 Danish women over a period of 30 years and concluded that the risk for breast cancer was 0.97% and it increased with age < 40 years at diagnosis of endometriosis. There is an increased risk association between endometriosis and breast cancer in postmenopausal patients with endometriosis due to common risk factors [19]. Hence such females should also be followed up in order to screen and diagnose breast cancer early.

## Conclusions

Although endometriosis is a rare condition in postmenopausal women, it can occur irrespective of age or location. Hence, a high degree of clinical suspicion is necessary to diagnose postmenopausal endometrial ovarian cysts before worsening potential complications or the chance of developing into a malignancy.
